# Development of a new equation in fuzzy logic analysis for ascertaining appropriate dose of gamma irradiation of virgin coconut oil

**DOI:** 10.1016/j.mex.2018.01.013

**Published:** 2018-02-01

**Authors:** Anupama Bose, Paramita Bhattacharjee

**Affiliations:** Department of Food Technology and Biochemical Engineering, Jadavpur University, Kolkata 700 032, India

**Keywords:** S, triplet, SO, overall sensory score, Q_REL_, relative weightage, Ya, defuzzified score under condition of (a + c) ≤ 100, Yaˊ, defuzzified score under condition of (a + c) > 100, B_x_, overall membership function, S_m_, similarity values, IVCO, irradiated virgin coconut oil, Deodorized coconut oil, Shelf-life study, Sensory evaluation, Fuzzy logic, New developed equation, Similarity values

## Abstract

Our previous investigation had established 4.2 kGy to be the appropriate dosage of gamma irradiation for removal of obnoxious rancid-acid-odor of virgin coconut oil (VCO) on the basis of sensory and electronic nose (e nose) studies. This study endeavored to revalidate the sensory data employing fuzzy logic analysis. An equation has been developed for the first time for deriving defuzzified scores, when the sum of the first and third coordinates of the triplet *(a b c)* of overall sensory score was greater than 100, i.e. (a + c) > 100. This study reaffirmed 4.2 kGy to be the most preferred dose for deodorization of VCO. Besides, ranking of the VCO samples were similar by either approach.

• According to the fuzzy logic method, overall sensory scores were assigned to the VCO samples under investigation, these sensory scores have been represented by a triangle and a polygon when (a+c) is less and more than100, respectively.
• The coordinates of the polygon were determined and a new equation has been developed for evaluating defuzzified scores, which has been validated by similarity value analysis.
• This new methodology of fuzzy logic analysis can be used to rank samples rapidly and reliably, without any complexity of conventional similarity value approach.

## Graphical abstract

**Figure:**
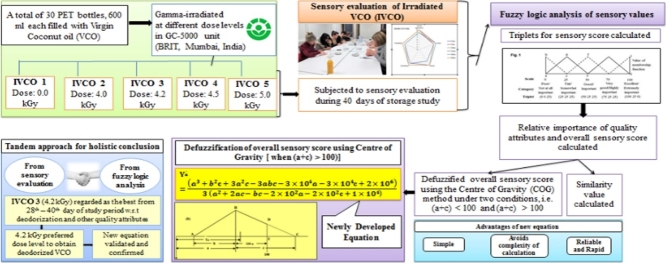

**Specifications Table**

**Table:** 

**Subject Area**	Agricultural and Biological Sciences
**More specific subject area:**	Food Technology
**Method name:**	Fuzzy logic analysis
**Name and reference of original method**	Fuzzy logic analysis H. Das, Sensory Evaluation Using Fuzzy Logic In Food Processing Operations Analysis, 2005, Asian Books, New Delhi, 383-402.
**Resource availability**	Hard copy of the above mentioned book

## Method details

### Background

Presently, quantification of food quality is being addressed in food research and business practices [1]. Consequently, data are essential to describe food qualities for product development, quality control and process control. Statistical techniques for sensory tests are routinely in use and are under continuous advancement. The data for statistical analysis are in linguistic expressions which must be converted by cumbersome processes to numerical values. This is circumvented by use of fuzzy logic theory, a mathematical technique which can directly quantify human linguistic expression and therefore enables quantification of primary and imprecise data obtained from sensory tests. This logical approach comprises of mainly three steps: i) an appropriate definition of how good a certain quality parameter level is, ii) a sound way to combine several quality parameters and iii) a way to express the overall quality based on all these individual parameters, considering their individual relative importance [2,5].

Studies from our previous investigation [3] have established 4.2 kGy to be the appropriate dosage of gamma irradiation for removal of obnoxious rancid-acid-odor of virgin coconut oil (VCO). This dosage was confirmed by sensory evaluation of irradiated oil samples and their odor profile analyses by e-nose. This study endeavors to establish concurrence of data obtained from e-nose analysis (of deodorized VCO conducted at a regular interval of seven days by thirty semi-trained panelists for a storage period of 40 days) and fuzzy logic analysis of sensory scores. The main objective of the current study was to develop an equation for deriving defuzzified scores when the sum of the first and third coordinates of the triplet (a b c) of overall sensory score (SO) is greater than 100, i.e. (a + c) > 100. The secondary objective of this work was to validate the new developed equation using the data obtained from similarity value and electronic nose(e-nose) analyses.

### Procurement of raw material

A gift sample of a leading brand of expeller pressed Virgin coconut oil (VCO) (obtained from coconut copra of *West coast tall* variety coconuts) with no added antioxidant and preservative was used in this study, in accordance with our previous study [3].

### Preparation of sample for irradiation

Thirty LDPE co-polymer screw capped bottles (500 mL) were sterilized under UV light in a laminar hood. Each bottle was filled with 500 mL VCO and was numbered serially. The bottled oil samples were then irradiated in a Co-60 γ irradiation chamber [(GC 5000; Serial No. GIC 038); source: Cobalt 60 solid (dose rate: 5.201 kGy h^−1^)] of BRIT, Mumbai in the National Instrument Laboratory (NIL) campus of Jadavpur University at 23 ± 2 °C at the pre-selected dose levels of 0.0 kGy (control set, IVCO 1), 4.0 kGy (IVCO 2), 4.2 kGy (IVCO 3), 4.5 kGy (IVCO 4) and 5.0 kGy (IVCO 5), in accordance with the procedure reported by [3]. Post irradiation, all samples were stored at room temperature (23 ± 2 °C) in these airtight LDPE co-polymer screw capped bottles for 40 days. Sensory evaluation was conducted for the irradiated oil samples at an interval of seven days for a total period of forty days in accordance with our previous report [3].

### Sensory evaluation of irradiated coconut oil samples

Sensory evaluation of the above irradiated coconut oil samples were conducted inside a university classroom at 24 ± 1 °C in bright light. The panel for sensory evaluation consisted of 30 members (in the age group of 21–45 years) including trained students and few semi-trained staff of the Department of Food Technology and Biochemical Engineering, Jadavpur University, Kolkata, India. The 30-member panel was sufficient for our analysis since fuzzy logic reportedly minimizes all the biasing effect, generated due to different consumers’ perception [5]. Panelists evaluated the samples in terms of aroma, color, homogeneity and flowability using the standard 5-point hedonic scale (1–5) [1 = not satisfactory, 2 = fair, 3 = medium, 4 = good, 5 = excellent]. During testing, the panelists were monitored by the authors. The panelists were served with coffee beans (for smelling) in between to enable them to correctly differentiate the aroma among the irradiated oil samples. The order of the samples served was same for all the panelists in a particular session, but varied between two sessions. Panelists were asked to provide numerical score against each sensory attribute of each sample and also give their preference against each quality attribute (aroma, color, homogeneity and flowability), following respective scale factors [not at all important (NI), somewhat important (SI), important (I), highly important (HI), extremely important (EI)] for each sample, relative to the entire sample set. The main steps of fuzzy modeling of sensory evaluation are: (1) calculation of triplet (S) corresponding to different quality attributes (aroma, color, homogeneity and flowability); (2) relative weightage (Q_REL_) of particular quality attributes (aroma, color, homogeneity and flowability), (3) overall sensory score (SO); (4) calculation of defuzzified score (Y_a_) while (a + c) < 100 and (Yá) when (a + c) > 100 and (5) ranking of quality attributes and grading of samples [5].

### Triangular fuzzy number (TFN) and fuzzy arithmetic operations

**Fig. 1:**
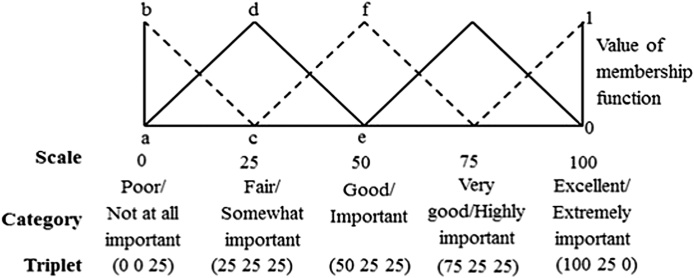
Distribution pattern of five point sensory scale.

Triangular membership function distribution pattern of sensory scale is shown in Fig. 1. This represents a 5-point sensory scale viz., poor/not at all important, fair/somewhat important, good/important, very good/highly important and excellent/extremely important. The triplet (a, b, c) associated with sensory scale is a triangular fuzzy number. Here, ‘a’ (first number of triplets) is called the mean value of the fuzzy number and it denotes the coordinate of abscissa at which the value of membership function is 1. The second and third numbers of the triplets ‘b’ and ‘c’ are called the left and right spreads respectively, whose membership functions are 0 [6].

### Triplets for sensory scores of irradiated oil samples and overall quality of samples

Triplets corresponding to specific quality attributes such as aroma, color, homogeneity and flowability were derived from the sum of sensory scores (Table 1) and the triplets associated with sensory scale and total number of panelists. For example, for IVCO 1 (0.0 kGy) on day 0: out of 30 panelists, 18 panelists agreed on a ‘not significant’ score, while 12 panelists provided the score ‘fair’ against the sensory attribute aroma (Table 1). Therefore, the triplets of the sensory scores for aroma of IVCO 1 (S1A) can be obtained as follows:

(1) $$S1A_{0}=\frac{18\left(0025\right)+12\left(252525\right)+0\left(502525\right)+0\left(752525\right)+0\left(100250\right)}{\left(18+12+0+0+0\right)}$$

**Table 1:** Panelists’ preference for specific quality attributes of irradiated coconut oil samples and corresponding triplets.

Sensory quality attributes of irradiated coconut oil samples	Days	Not satisfactory	Fair	Medium	Good	Excellent	Triplets of sensory score
Aroma							
IVCO 1	0	18	12	0	0	0	S1A_0_ = (10.00 10.00 25.00)
	7	16	14	0	0	0	S1A_7_ = (11.67 11.67 25.00)
	14	18	12	0	0	0	S1A_14_ = (10.00 10.00 25.00)
	21	19	11	0	0	0	S1A_21_ = (9.17 9.17 25)
	28	20	10	0	0	0	S1A_28_ = (8.33 8.33 25.00)
	40	20	10	0	0	0	S1A_40_ = (8.33 8.33 25.00)

IVCO 2	0	12	18	0	0	0	S2A_0_ = (15.00 15.00 25.00)
	7	15	15	0	0	0	S2A_7_ = (12.50 12.50 25.00)
	14	17	13	0	0	0	S2A_14_ = (10.83 10.83 25.00)
	21	18	12	0	0	0	S2A_21_ = (10.00 10.00 25.00)
	28	19	11	0	0	0	S2A_28_ = (9.17 9.17 25.00)
	40	20	10	0	0	0	S2A_40_ = (8.33 8.33 25.00)

IVCO 3	0	0	17	13	0	0	S3A_0_ = (35.83 25.00 25.00)
	7	0	11	19	0	0	S3A_7_ = (40.83 25.00 25.00)
	14	0	10	20	0	0	S3A_14_ = (41.67 25.00 25.00)
	21	0	8	22	0	0	S3A_21_ = (43.33 25.00 25.00)
	28	0	0	0	7	23	S3A_28_ = (94.17 25.00 5.83)
	40	0	0	0	1	29	S3A_40_ = (99.17 25.00 0.83)

IVCO 4	0	0	16	14	0	0	S4A_0_ = (36.67 25.00 25.00)
	7	0	21	9	0	0	S4A_7_ = (32.50 25.00 25.00)
	14	0	30	0	0	0	S4A_14_ = (25.00 25.00 25.00)
	21	17	13	0	0	0	S4A_21_ = (10.83 10.83 25.00)
	28	18	12	0	0	0	S4A_28_ = (10.00 10.00 25.00)
	40	19	11	0	0	0	S4A_40_ = (9.17 9.17 25.00)

IVCO 5	0	0	17	13	0	0	S5A_0_ = (35.83 25.00 25.00)
	7	0	24	6	0	0	S5A_7_ = (30.00 25.00 25.00)
	14	0	30	0	0	0	S5A_14_ = (25.00 25.00 25.00)
	21	18	12	0	0	0	S5A_21_ = (10.00 10.00 25.00)
	28	19	11	0	0	0	S5A_28_ = (9.17 9.17 25.00)
	40	20	10	0	0	0	S5A_40_ = (8.33 8.33 25.00)

Colour							
IVCO 1	0	0	17	13	0	0	S1C_0_ = (35.83 25.00 25.00)
	7	0	18	12	0	0	S1C_7_ = (35.00 25.00 25.00)
	14	0	17	13	0	0	S1C_14_ = (35.83 25.00 25.00)
	21	18	12	0	0	0	S1C_21_ = (10.00 10.00 25.00)
	28	19	11	0	0	0	S1C_28_ = (9.17 9.17 25.00)
	40	19	11	0	0	0	S1C_40_ = (9.17 9.17 25.00)

IVCO 2	0	0	19	11	0	0	S2C_0_ = (34.17 25.00 25.00)
	7	0	19	11	0	0	S2C_7_ = (34.17 25.00 25.00)
	14	0	18	12	0	0	S2C_14_ = (35.00 25.00 25.00)
	21	11	19	0	0	0	S2C_21_ = (15.83 15.83 25.00)
	28	12	18	0	0	0	S2C_28_ = (15.00 15.00 25.00)
	40	12	18	0	0	0	S2C_40_ = (15.00 15.00 25.00)

IVCO 3	0	0	18	12	0	0	S3C_0_ = (35.00 25.00 25.00)
	7	0	13	17	0	0	S3C_7_ = (39.17 25.00 25.00)
	14	0	12	18	0	0	S3C_14_ = (40.00 25.00 25.00)
	21	0	5	20	5	0	S3C_21_ = (50.00 25.00 25.00)
	28	0	0	5	15	5	S3C_28_ = (62.50 20.83 16.67)
	40	0	0	0	2	28	S3C_40_ = (98.33 25.00 1.67)

IVCO 4	0	0	16	14	0	0	S4C_0_ = (36.67 25.00 25.00)
	7	0	11	19	0	0	S4C_7_ = (40.83 25.00 25.00)
	14	0	11	19	0	0	S4C_14_ = (40.83 25.00 25.00)
	21	0	13	17	0	0	S4C_21_ = (39.17 25.00 25.00)
	28	0	14	16	0	0	S4C_28_ = (38.33 25.00 25.00)
	40	0	15	15	0	0	S4C_40_ = (37.50 25.00 25.00)

IVCO 5	0	0	17	13	0	0	S5C_0_ = (35.83 25.00 25.00)
	7	0	10	20	0	0	S5C_7_ = (41.67 25.00 25.00)
	14	0	15	5	0	0	S5C_14_ = (20.83 16.67 16.67)
	21	0	13	17	0	0	S5C_21_ = (39.17 25.00 25.00)
	28	0	14	16	0	0	S5C_28_ = (38.33 25.00 25.00)
	40	0	15	15	0	0	S5C_40_ = (37.50 25.00 25.00)

Homogeneity							
IVCO 1	0	0	15	15	0	0	S1H_0_ = (37.50 25.00 25.00)
	7	0	15	15	0	0	S1H_7_ = (37.50 25.00 25.00)
	14	0	18	12	0	0	S1H_14_ = (35.00 25.00 25.00)
	21	0	13	17	0	0	S1H_21_ = (39.17 25.00 25.00)
	28	0	13	17	0	0	S1H_28_ = (39.17 25.00 25.00)
	40	0	13	17	0	0	S1H_40_ = (39.17 25.00 25.00)

IVCO 2	0	0	19	11	0	0	S2H_0_ = (34.17 25.00 25.00)
	7	0	16	14	0	0	S2H_7_ = (36.67 25.00 25.00)
	14	0	17	13	0	0	S2H_14_ = (35.83 25.00 25.00)
	21	0	19	11	0	0	S2H_21_ = (34.17 25.00 25.00)
	28	0	21	9	0	0	S2H_28_ = (32.50 25.00 25.00)
	40	0	21	9	0	0	S2H_40_ = (32.50 25.00 25.00)

IVCO 3	0	0	14	16	0	0	S3H_0_ = (38.83 25.00 25.00)
	7	0	13	17	0	0	S3H_7_ = (39.17 25.00 25.00)
	14	0	14	16	0	0	S3H_14_ = (38.33 25.00 25.00)
	21	0	7	19	4	0	S3H_21_ = (47.50 25.00 25.00)
	28	0	0	5	25	0	S3H_28_ = (70.83 25.00 20.00)
	40	0	0	0	3	27	S3H_40_ = (98.33 25.00 25.00)

IVCO 4	0	0	17	13	0	0	S4H_0_ = (35.83 25.00 25.00)
	7	0	15	15	0	0	S4H_7_ = (37.50 25.00 25.00)
	14	0	11	19	0	0	S4H_14_ = (40.83 25.00 25.00)
	21	0	13	17	0	0	S4H_21_ = (39.17 25.00 25.00)
	28	0	14	16	0	0	S4H_28_ = (38.33 25.00 20.00)
	40	0	15	15	0	0	S4H_40_ = (37.50 25.00 25.00)

IVCO 5	0	0	16	14	0	0	S5H_0_ = (36.67 25.00 25.00)
	7	0	16	14	0	0	S5H_7_ = (36.67 25.00 25.00)
	14	0	14	16	0	0	S5H_14_ = (38.33 25.00 25.00)
	21	0	13	17	0	0	S5H_21_ = (39.17 25.00 25.00)
	28	0	14	16	0	0	S5H_28_ = (38.33 25.00 20.00)
	40	0	15	15	0	0	S5H_40_ = (37.50 25.00 25.00)

Flowability							
IVCO 1	0	0	12	18	0	0	S1F_0_ = (40.00 25.00 25.00)
	7	0	17	13	0	0	S1F_7_ = (35.83 25.00 25.00)
	14	0	16	14	0	0	S1F_14_ = (36.67 25.00 25.00)
	21	0	14	16	0	0	S1F_21_ = (38.33 25.00 25.00)
	28	0	14	16	0	0	S1F_28_ = (38.33 25.00 25.00)
	40	0	14	16	0	0	S1F_40_ = (38.33 25.00 25.00)

IVCO 2	0	0	15	15	0	0	S2F_0_ = (37.50 25.00 25.00)
	7	0	18	12	0	0	S2F_7_ = (35.00 25.00 25.00)
	14	0	15	15	0	0	S2F_14_ = (37.50 25.00 25.00)
	21	0	14	16	0	0	S2F_21_ = (38.33 25.00 25.00)
	28	0	15	15	0	0	S2F_28_ = (37.50 25.00 25.00)
	40	0	15	15	0	0	S2F_40_ = (37.50 25.00 25.00)

IVCO 3	0	0	13	17	0	0	S3F_0_ = (39.17 25.00 25.00)
	7	0	15	15	0	0	S3F_7_ = (37.50 25.00 25.00)
	14	0	14	16	0	0	S3F_14_ = (38.33 25.00 25.00)
	21	0	7	20	3	0	S3F_21_ = (46.67 25.00 25.00)
	28	0	0	16	14	0	S3F_28_ = (61.67 25.00 25.00)
	40	0	0	0	10	20	S3F_40_ = (91.67 25.00 8.33)

IVCO 4	0	0	18	12	0	0	S4F_0_ = (35.00 25.00 25.00)
	7	0	18	12	0	0	S4F_7_ = (35.00 25.00 25.00)
	14	0	18	12	0	0	S4F_14_ = (35.00 25.00 25.00)
	21	0	14	16	0	0	S4F_21_ = (38.33 25.00 25.00)
	28	0	15	15	0	0	S4F_28_ = (37.50 25.00 25.00)
	40	0	16	14	0	0	S4F_40_ = (36.67 25.00 25.00)

IVCO 5	0	0	20	10	0	0	S5F_0_ = (33.33 25.00 25.00)
	7	0	18	12	0	0	S5F_7_ = (35.00 25.00 25.00)
	14	0	19	11	0	0	S5F_14_ = (34.17 25.00 25.00)
	21	0	15	15	0	0	S5F_21_ = (37.50 25.00 25.00)
	28	0	14	16	0	0	S5F_28_ = (38.33 25.00 25.00)
	40	0	15	15	0	0	S5F_40_ = (37.50 25.00 25.00)

Similar values for other quality attributes of all samples (during the entire storage period) were obtained using the aforesaid equation (Eq. (1)). Relative weightage (Q_REL_) for all sensory attributes (aroma, color, homogeneity and flowability) were calculated by dividing the triplets of each quality attribute with Q_SUM_ as has been described by Das [2] (Table 2). The triplets of overall sensory scores of all irradiated oil samples for each quality attribute were evaluated using Eq. (2). For example, the overall sensory score of IVCO 1 at day 0 (i.e., SO1_0_) (Table 4) would be obtained as follows:

(2) SO1_0_=S1A_0_ ⊙QA_REL_S1C_0_ ⊙ QC_REL_S1H_0_ ⊙ QH_REL_S1F_0_ ⊙ QF_REL_

where, S1A_0_,S1C_0_, S1H_0_ and S1F_0_ represent the triplets corresponding to aroma, color, homogeneity and flowability of IVCO 1 at day 0, respectively. QA_REL_, QC_REL_, QH_REL_ and QF_REL_ signify the triplets corresponding to relative weightages of aroma, color, homogeneity and flowability, of irradiated oil samples, respectively. In this way, the overall sensory scores for all samples (during storage period) were calculated.

**Table 2:** Panelists’ preference for quality attributes of irradiated coconut oil samples in general and corresponding triplets.

Quality attributes	NI	SI	I	HI	EI	Triplets of sensory score	triplets of relative weightage
Aroma	0	0	0	0	30	(75.00 25.00 25.00)	(0.50 0.17 0.17)
Colour	30	0	0	0	0	(0 0 25.00)	(0.00 0.00 0.17)
Homogeneity	0	0	30	0	0	(50.00 25.00 25.00)	(0.33 0.17 0.17)
Flowability	0	0	0	0	0	(25.00 25.00 25.00)	(0.17 0.17 0.17)

Triplets (a, b, c) representing the overall sensory score (SO) was denoted by triangle XYZ (Fig. 3a and b). When the value of (a + c) ≤ 100, the triangle XYZ lies within the sensory scale interval [0,100]. This is represented by Fig. 3(a). Under this condition, the value of Y_a_ in terms of a, b and c can be expressed by Eq. (3) [7], where Y_a_ is the centroid as well as the defuzzified score of triangle XYZ (Fig. 3a).

(3) $$\text{Ya}=\frac{\left(3\text{a}-\text{b}+\text{c}\right)}{3}$$

However, under the second condition, i.e. (a + c) > 100, polygon XYMN has to be considered instead of triangle XYZ, to maintain the standard sensory scale in the range 0–100 (Fig. 3b). This necessitated development of a new equation for deriving the defuzzified score of a sample when (a + c) > 100.

### Development of new equation for obtaining defuzzified scores

Eq. (3) was not considered for finding the value of defuzzified scores of polygon XYMN [condition: (a + c) > 100]. It is well known that the centroid of the polygon represents the defuzzified score of the same. Here, centroid of polygon XYMN was derived using Eq. (4) which represents centroid of a polygon in general.

(4) $$\text{Y}á=\frac{1}{6A}\sum_{i=0}^{n-1}\left(x_{i}+x_{i+1}\right)\left({\text{x}}_{\text{i}}{\text{y}}_{\text{i}+1}+{\text{x}}_{\text{i}+1}{\text{y}}_{\text{i}}\right)$$

As per Fig. 3(b), Eq. (4) can be written in the following way:

(5) $$\text{Y}á=\frac{1}{6\text{A}}\left[\left({\text{x}}_{0}+{\text{x}}_{1}\right)\left({\text{x}}_{0}{\text{y}}_{1}-{\text{x}}_{1}{\text{y}}_{0}\right)+\left({\text{x}}_{1}+{\text{x}}_{2}\right)\left({\text{x}}_{1}{\text{y}}_{2}-{\text{x}}_{2}{\text{y}}_{1}\right)+\left({\text{x}}_{2}+{\text{x}}_{3}\right)\left({\text{x}}_{2}{\text{y}}_{3}-{\text{x}}_{3}{\text{y}}_{2}\right)\right]$$

where,

(6) $$A=\frac{1}{2}[\left({\text{x}}_{0}{\text{y}}_{1}-{\text{x}}_{1}{\text{y}}_{0}\right)+\left({\text{x}}_{1}{\text{y}}_{2}-{\text{x}}_{2}{\text{y}}_{1}\right)+\left({\text{x}}_{2}{\text{y}}_{3}-{\text{x}}_{3}{\text{y}}_{2}\right)]$$

It therefore follows that

(7) $$∴\text{Y}á=\frac{\left({\text{x}}_{0}+{\text{x}}_{1}\right)\left({\text{x}}_{0}{\text{y}}_{1}-{\text{x}}_{1}{\text{y}}_{0}\right)+\left({\text{x}}_{1}+{\text{x}}_{2}\right)\left({\text{x}}_{1}{\text{y}}_{2}-{\text{x}}_{2}{\text{y}}_{1}\right)+\left({\text{x}}_{2}+{\text{x}}_{3}\right)\left({\text{x}}_{2}{\text{y}}_{3}-{\text{x}}_{3}{\text{y}}_{2}\right)}{3\left({\text{x}}_{0}{\text{y}}_{1}-{\text{x}}_{1}{\text{y}}_{0}\right)+\left({\text{x}}_{1}{\text{y}}_{2}-{\text{x}}_{2}{\text{y}}_{1}\right)+\left({\text{x}}_{2}{\text{y}}_{3}-{\text{x}}_{3}{\text{y}}_{2}\right)}$$

Here, Yá and A are the centroid and area of polygon XYMN, respectively. In Fig. 3b, the coordinates of points X, Y, M, N are (x_0_ y_0_), (x_1_ y_1_) (x_2_ y_2_) (x_3_ y_3_), respectively. In accordance with Fig. 3b, the values of these coordinates are as follows: x_0_ = (a–b), x_1_ = a, x_2_ = x_3_ = 100 and y_0_ = y_3_ = 0, y_1_ = 1, y_2_ = MN. The MN value was obtained from the rule of similarity of triangle, discussed below. Here, Δ YOZ and Δ MNZ are always right angled triangles. Therefore, the rule of similarity was employed here and values of MN were obtained (shown below). The centroid of polygon XYMN was found by putting all the values of coordinates in Eq. (7). Thus final equation of Yá of polygon XYMN has been derived and reported by us for the first time and is represented by Eq. (8). This equation is not reported in literature.

(8) $$\begin{array}{c} \text{MN}/\text{YO}=\text{ NZ}/\text{OZ}\\ ∴\text{MN}=\frac{\text{a}}{\text{c}}+1-\frac{100}{\text{c}}\\ ∴\text{A}=\frac{2ac-bc+a^{2}+200a-200c+10000}{c}\\ ∴Y\overset{´}{a}=\frac{\left(a^{3}+b^{2}c+3a^{2}c-3abc-3\times 10^{4}a-3\times 10^{4}c+2\times 10^{6}\right)}{3\left(a^{2}+2ac-bc-2\times 10^{2}a-2\times 10^{2}c+1\times 10^{4}\right)} \end{array}$$

### Defuzzified values for ranking of samples and their quality attributes

**Fig 2:**
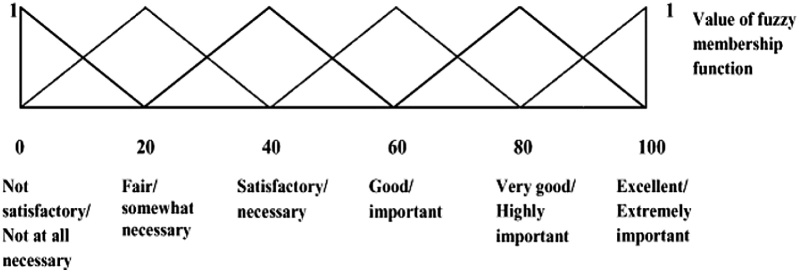
Standard Fuzzy scale.

The relative importance of four quality attributes (*viz*., aroma, color, homogeneity and flowability) was obtained from their defuzzified scores. The defuzzified scores were compared with the six point sensory scales of linguistic parameters, i.e., not satisfactory, fair, satisfactory, good, very good and excellent (Fig. 2), which are classified by following number ranges: 10, 10–30, 30–50, 50–70, 70–90, 90–100, respectively. In order to judge all the samples on each day of storage with respect to all quality attributes (aroma, color, homogeneity and flowability), the defuzzified scores were considered in this comparative study, instead of S_m_. The defuzzified scores of all samples were calculated using Eq. (3) or Eq. (8), depending on the value of (a + c).

It was observed that, (a + c) values of IVCO 3 on days 21_,_ 28 and 40 were 101.53, 144.58 and 164.44, respectively [i.e. (a + c) > 100]. For these three cases, Eq. (8) was used instead of Eq. (3) for obtaining their respective defuzzified scores.

### Shortcoming of the procedure for ranking of the samples by similarity principle

**Fig. 3:**
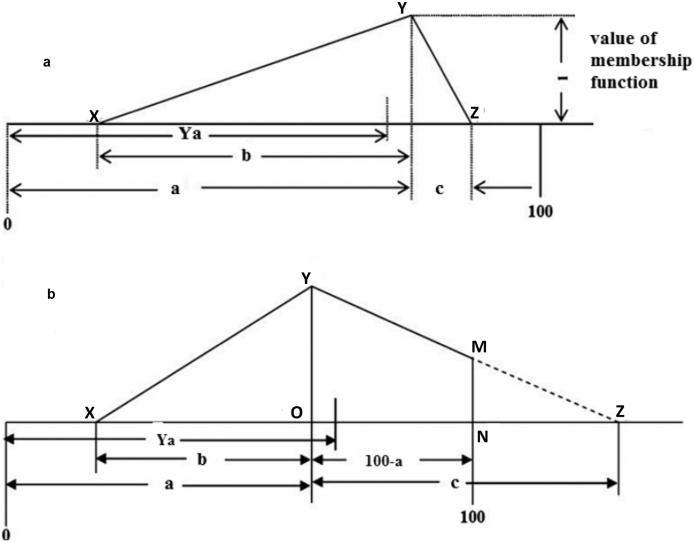
3 (a) Graphical view of overall sensory score as triangle ABC when the value of (a+c) is less than 100, the triangle XYZ lies within the sensory scale interval [0, 100], (b) Graphical view of overall sensory score as polygon XYMN when the value of (a+c) is greater than 100, a part of the triangle XYZ lies beyond the interval 0 to 100.

The overall sensory score (obtained as a single triplet) was fitted into the six point sensory scale (referred to as standard fuzzy scale) employing similarity analysis. Calculation of S_m_ is very time consuming owing to complexity of its calculation. S_m_ for the irradiated oil samples were calculated employing overall membership function values of sensory scores and values of membership functions of standard fuzzy scale. Standard fuzzy scale, viz. not satisfactory/not at all necessary, fair/somewhat necessary, medium/necessary, good/important, very good/highly important and excellent/extremely important was designated as F1, F2, F3, F4, F5 and F6, respectively (Fig. 3). As per Fig. 3, the values of membership functions are defined by a set of 10 numbers, elaborated in (9).

(9) $$\begin{array}{c} \begin{array}{c} F1=\left(1,0.5,0,0,0,0,0,0,0,0\right)\\ F2=\left(1,0.5,0,0,0,0,0,0,0,0\right)\\ F3=\left(0,0,0.5,1,1,0.5,0,0,0,0\right) \end{array}\\ F4=\left(0,0,0,0,0.5,1,1,0.5,0,0\right)\\ F5=\left(0,0,0,0,0,0,0.5,1,1,0.5\right)\\ F6=\left(0,0,0,0,0,0,0,0,0.5,1\right) \end{array}$$

The membership function of a triplet (a b c) has been presented graphically in Fig. 3. According to Fig. 3, for the triplet (a b c), the value of membership function is 1 when the value of abscissa is ‘a’ and the value of membership function is 0 when abscissa is less than ‘(a–b)’ or greater than ‘(a + c)’. The value of membership function, B_x_ at x = 0, 10, 20, 30, 40, 50, 60, 70, 80, 90 and 100 obtained from overall sensory scores of the irradiated coconut oil samples were calculated from Eq. (10). After calculating the B_x_ values (as a set of ten values) for each sample, S_m_ were derived using Eq. (11). Determination of S_m_ comprises of the following steps: step1: determination of B_x_ values for each sample; step 2: multiplication of matrix F with transpose matrix of F (F х F^T^); step 3: multiplication of matrix F with transpose matrix of B (F х B_x_^T^) and step 4: multiplication of matrix B with transpose matrix of B (B_x_ х B_x_^T^). Thus S_m_ values were calculated under six categories of sensory scales, employing these steps. The highest S_m_ was considered and its corresponding category described the quality of sample. This approach clearly demonstrated that determination of defuzzified scores using Eq. (8) is relatively simpler than determination of S_m_ values. Thus the advantage of using Eq. (8) [condition: (a + c) > 100] lies in its simplicity.

(10) $$\begin{array}{c} {\text{B}}_{\text{X}}=\frac{X-\left(a-b\right)}{b}\;\;\text{ for}\left(a-b\right)<x<a\\ {\text{B}}_{\text{x}}=\frac{\left(a+c\right)-x}{c}\;\;\text{ for}a<x<\left(a+c\right)\\ {\text{B}}_{\text{x}}=0\text{for}x<(a-b)\;\text{and}\;\;\;x>(a+c) \end{array}$$

(11) $$S_{m}(F,B)=\frac{\text{F}\times {\text{B}}^{\text{T}}}{\text{Max}\left(\text{F}\times {\text{F}}^{\text{T}}\text{and}\text{B}\times {\text{B}}^{\text{T}}\right)}$$

### Validation of the newly developed equation

The conclusion derived from the results of newly developed equation (Eq. (8)) was validated from results of similarity value and also by odor profile analysis of the irradiated oil samples using e-nose(ENOVISION, C-DAC). The signal responses (ΔR/R) of the irradiated coconut oil samples, obtained from e-nose analysis have been reported by our research group [3]. Validation by S_m_ was carried out by standard fuzzy and sensory scales.

### Ranking of quality attributes of irradiated coconut oil samples based on defuzzified scores

The triplets of sensory scores for four quality attributes (aroma, color, homogeneity and flowability) in general, were calculated using Eq. (1). Sensory scores, triplets associated with these scores of quality attributes of irradiated coconut oil samples and relative weightages of all quality attributes are presented in Table 2. Defuzzified scores for four quality attributes of each sample were calculated using Eq. (3) (Table 3). The Y_a_ values for aroma and homogeneity were obtained as 75.00 and 50.00, respectively. These results illustrated that aroma had received the highest importance in sensory acceptance followed by sample homogeneity. The defuzzified scores for other two quality attributes (color and flowability) were 8.33 and 25, respectively. Therefore, the following trend of preference of quality attributes of the irradiated coconut oil samples can be arrived at using linguistic representations of standard sensory scale.

**Table 3:** Defuzzified score of quality attributes of irradiated coconut oil samples.

Quality attributes	Q (for relative importance)			Defuzzified scores
	a	b	c	
Aroma	75	25	25	75.00
Colour	0	0	25	8.33
Homogeneity	50	25	25	50.00
Flowability	25	25	25	25.00

Aroma (highly important) > homogeneity (important) > flowability (somewhat important) > color (not at all important)

### Defuzzified scores of deodorized coconut irradiated oil samples and sample ranking

The sensory scores of five samples w.r.t four quality attributes (aroma, color, homogeneity and flowability) on each day of storage have been presented in Table 1.The triplets associated with sensory scores were determined using Eq. (1). Defuzzified scores for the stored irradiated-coconut oil samples were evaluated using Eq. (3) or Eq. (8), depending on the value of (a + c). For example, triplets of overall sensory score of IVCO 1 on day 0 (SO1_0_) were found to be (24.57 32.08 45.56) from Eq. (2). Here, (a + c) = 70.13 (<100). Therefore, Eq. (3) was employed for finding defuzzified scores of IVCO 1 on day 0. For IVCO 3, Eq. (3) was applicable up to day 14. From day 21 onwards, (a + c) values were greater than 100. Triplets of overall sensory score (SO3_21_) of IVCO 3 on day 21 were (45.28 47.92 56.25). The value of (a + c) was 101.53 (>100). Therefore, under this condition, Eq. (8) was used instead of Eq. (3) to determine the defuzzified scores (Yá) of IVCO 1.

**Fig 4:**
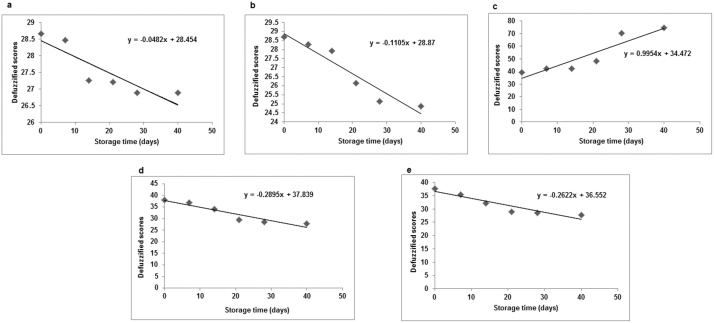
Plot of defuzzified scores of irradiated coconut oil samples with storage time (days) for (a) IVCO 1(0 kGy), (b) IVCO 2 (4.0 kGy), (c) IVCO 3 (4.2 kGy), (d) IVCO 4 (4.5 kGy), and (e) IVCO 5 (5.0 kGy).

In case of IVCO 1 and 2, defuzzified scores (Y_a_) declined gradually up to day 40 (Table 4), meaning that sample acceptance with respect to all quality attributes deteriorated with time. Y_a_ values of IVCO 1 (27.22) and 2 (26.16) were found to be maximum on day 21 of storage period. It implied that the above two samples reached almost saturation level of deterioration on the said day. For IVCO 4 and 5, the defuzzified scores declined in a similar manner as those of IVCO 1 and 2 with a variation in slope (Fig. 4). From Table 4, it was found that the defuzzified scores of IVCO 4 and 5 were obtained under category ‘satisfactory’ up to day 14, which came down to 29.40 and 28.98, respectively on day 21 under category ‘fair’. Therefore, 4.5 kGy and 5.0 kGy dose levels did not show effective deodorization effects in irradiated coconut oil samples with time, especially with respect to its rancid-acid aroma. Comparing the defuzzified scores of all the five samples on each day of storage study, it was observed that only IVCO 3 underwent continuous improvement with time, implying that 4.2 kGy dose level had effectively deodorized the oil sample with time. The defuzzified scores for IVCO 3 became high from day 28 onwards, i.e. 70.21 on day 28 and 74.70 on day at 40, respectively (both under ‘very good’ category). The defuzzified scores for the rest of the samples were found to be below 50 throughout the study period and therefore they were under categories of ‘fair’ or ‘satisfactory’. Thus, it was established that 4.2 kGy irradiated coconut oil sample alone had good sensory acceptance specifically with respect to deodorization (aroma) from day 28 onwards as had been inferred from our studies using e-nose(discussed later).

**Table 4:** Defuzzified scores of irradiated coconut oil sample.

Time	Sample no.	SO			Y_a_/Y_a ´_	
		a	b	c		Remarks
Day 0	IVCO 1	24.17	32.08	45.56	28.66	Fair
	IVCO 2	25.14	34.44	45.14	28.70	Fair
	IVCO 3	37.22	43.89	49.72	39.17	Satisfactory
	IVCO 4	36.11	42.92	49.03	38.15	Satisfactory
	IVCO 5	35.69	42.64	48.61	37.69	Satisfactory

Day 7	IVCO 1	24.31	32.50	45.00	28.47	Fair
	IVCO 2	24.31	32.78	44.72	28.29	Fair
	IVCO 3	39.74	45.26	52.01	41.99	Satisfactory
	IVCO 4	34.58	42.50	49.31	36.85	Satisfactory
	IVCO 5	33.06	41.94	48.89	35.57	Satisfactory

Day 14	IVCO 1	22.78	31.11	44.58	27.27	Fair
	IVCO 2	23.61	31.94	44.86	27.92	Fair
	IVCO 3	40.00	44.72	51.39	42.22	Satisfactory
	IVCO 4	31.94	41.81	48.61	34.21	Satisfactory
	IVCO 5	30.97	41.25	44.72	32.13	Satisfactory

Day 21	IVCO 1	24.03	31.53	41.11	27.22	Fair
	IVCO 2	22.78	31.25	41.39	26.16	Fair
	IVCO 3	45.28	47.92	56.25	**48.04**	Satisfactory
	IVCO 4	24.86	32.64	46.25	29.40	Fair
	IVCO 5	24.31	31.94	45.97	28.98	Fair

Day 28	IVCO 1	23.61	30.97	40.83	26.90	Fair
	IVCO 2	21.67	30.28	40.69	25.14	Fair
	IVCO 3	80.97	62.78	63.61	**70.21**	*Very good*
	IVCO 4	24.03	31.81	45.69	28.66	Fair
	IVCO 5	23.75	31.39	45.69	28.52	Fair

Day 40	IVCO 1	23.61	30.97	40.83	26.90	Fair
	IVCO 2	21.25	29.72	40.56	24.86	Fair
	IVCO 3	97.36	73.06	67.08	**74.71**	*Very good*
	IVCO 4	23.19	30.97	45.14	27.92	Fair
	IVCO 5	22.92	30.56	45.14	27.78	Fair

The bold values in Table 4 signify that defuzzified scores of IVCO 3 were found to exceptionally increase from day 21 compared to the rest of the samples (IVCO 1, 2, 4 and 5). In addition, the summation of first and third coordinates of overall sensory score of IVCO 3 was found to be greater than 100 from day 21 onwards until day 40.

### Validation of new developed equation by similarity principle

Values of overall membership function (B_x_) and similarity values of five samples (IVCO −1, 2, 3, 4, 5) on each storage day were calculated using Eqs. (10) and (11), respectively. For example, the triplet of overall sensory score of IVCO 1 at day 0 was found as (24.57 32.08 45.56), i.e. a = 24.57, b = 32.08, c = 45.56. Using the value of this triplet in Eq. (10), the values of membership function for IVCO 1(at day 0) was obtained as B1_0_ = (0.5582 0.8700 1 0.8720 0.6525 0.4330 0.2135 0.0000 0.0000 0.0000). Similar values for other samples on each day were evaluated using Eq. (10) and is presented in Table 5. Subsequently, S_m_ values of all five samples (IVCO-1, 2, 3, 4, 5) were calculated using Eq. (11) (Table 6). The S_m_ values of both IVCO 1 and 2 were under “satisfactory” category throughout the period of the study. The similar trend was followed by IVCO 4 and 5 from day 21. IVCO 3 had the highest S_m_ under “good” category up to day 21. However, on day 28, S_m_ value was found to be maximum and therefore attested the VCO sample to “very good” category which remained unaltered until the last day of the study (day 40). These findings (obtained from defuzzified scores and S_m_ analyses) revealed that IVCO 3, i.e. VCO irradiated at 4.2 kGy had no objectionable odor and therefore was sensorically highly accepted. This was in agreement with our previous findings [3] and we had reported that deodorization in this sample was owing to radiolysis of octanoic acid in the same.

**Table 5:** Overall membership function values of irradiated coconut oil samples.

Storage day	Values of overall membership functions (B_x_)									
Day 0										
IVCO 1	0.5582	0.8700	1	0.8720	0.65254	0.4330	0.2135	0	0	0
IVCO 2	0.6001	0.9273	1	0.8238	0.5974	0.3710	0.1446	0	0	0
IVCO 3	0.3798	0.6076	0.8354	1	0.9440	0.7430	0.5418	0.3407	0.1395	0
IVCO 4	0.3916	0.6246	0.8576	1	0.9206	0.7170	0.5127	0.3087	0.1048	0
IVCO 5	0.3975	0.6320	0.8665	1	0.9113	0.7060	0.4998	0.2941	0.0884	0

Day 7										
IVCO 1	0.5597	0.8674	1.1751	0.8736	0.6513	0.4291	0.2069	0	0	0
IVCO 2	0.5635	0.8685	1.1736	0.8728	0.6492	0.4255	0.2019	0	0	0
IVCO 3	0.3578	0.5802	0.8025	1.1717	0.9783	0.7850	0.5916	0.3983	0.2049	0.0116
IVCO 4	0.3750	0.6058	0.8366	1.1412	0.9418	0.7423	0.5429	0.3434	0.1440	0
IVCO 5	0.3566	0.5859	0.8152	1.1594	0.9616	0.7638	0.5661	0.3683	0.1705	0

Day 14										
IVCO 1	0.5892	0.9106	1.2321	0.8380	0.6137	0.3894	0.1651	0	0	0
IVCO 2	0.5739	0.8870	1.2001	0.8576	0.6346	0.4117	0.1888	0	0	0
IVCO 3	0.3659	0.5930	0.8201	1.1575	0.9586	0.7597	0.5609	0.3620	0.1631	0
IVCO 4	0.3428	0.5692	0.7956	1.1772	0.9810	0.7848	0.5886	0.3924	0.1962	0
IVCO 5	0.3418	0.5696	0.7974	1.1744	0.9782	0.7820	0.5858	0.3896	0.1934	0

Day 21										
IVCO 1	0.5550	0.8721	1	0.8547	0.6115	0.3683	0.1250	0	0	0
IVCO 2	0.5910	0.9110	1	0.8255	0.5839	0.3424	0.1007	0	0	0
IVCO 3	0.2637	0.4724	0.6811	0.8898	1	0.9161	0.7383	0.5605	0.3828	0.2049
IVCO 4	0.5447	0.8511	1	0.8888	0.6726	0.4564	0	0	0	0
IVCO 5	0.5519	0.8650	1	0.8762	0.6586	0.4412	0	0	0	0

Day 28										
IVCO 1	0.5605	0.8834	1	0.8435	0.5986	0.3537	0.1087	0	0	0
IVCO 2	0.6146	0.9448	1	0.7953	0.5495	0.3038	0.0580	0	0	0
IVCO 3	0	0.0288	0.1881	0.3474	0.5067	0.6660	0.8253	0.9845	1.1438	0.8580
IVCO 4	0.5589	0.8733	1	0.8693	0.6505	0.4316	0.2127	0	0	0
IVCO 5	0.5620	0.8805	1	0.8632	0.6443	0.4255	0.2066	0	0	0

Day 40										
IVCO 1	0.5605	0.8834	1.2063	0.8435	0.5986	0.3537	0.1087	0	0	0
IVCO 2	0.6215	0.9579	1.2944	0.7843	0.5377	0.2912	0.0446	0	0	0
IVCO 3	0	0	0.0780	0.2149	0.3518	0.4886	0.6255	0.7624	0.8993	1.1097
IVCO 4	0.5741	0.8970	1.2199	0.8491	0.6276	0.4061	0.1845	0	0.0000	0
IVCO 5	0.5772	0.9045	1.2317	0.8432	0.6216	0.4001	0.1786	0	0.0000	0

**Table 6:** Similarity values of irradiated oil samples and their ranking.

Sample no.	Storage day	Six categories					
		Not significant	Fair	Satisfactory	Good	Very good	Excellent
IVCO 1	0	0.0000	0.3394	*1.0398*	0.9010	0.3914	0.0430
	7	0.0000	0.4011	*1.0428*	0.9040	0.3878	0.0420
	14	0.0000	0.4367	*1.0965*	0.8968	0.3598	0.0343
	21	0.0000	0.4303	*1.1190*	0.9340	0.3469	0.0269
	28	0.0000	0.4420	*1.1403*	0.9352	0.3361	0.0238
	40	0.0000	0.4420	*1.1403*	0.9352	0.3361	0.0238

IVCO 2	0	0.0000	0.4522	*1.1215*	0.8959	0.3464	0.0306
	7	0.0000	0.4032	*1.0457*	0.9038	0.3850	0.0451
	14	0.0000	0.4167	*1.0655*	0.9001	0.3762	0.0385
	21	0.0000	0.4610	*1.1543*	0.9171	0.3239	0.0220
	28	0.0000	0.4603	*1.2100*	0.9119	0.2909	0.0132
	40	0.0000	0.5088	*1.2303*	0.9103	0.2794	0.0102

IVCO 3	0	0.0000	0.2074	0.6471	*0.8290*	0.5846	0.1856
	7	0.0000	0.1879	0.5976	*0.8037*	0.5987	0.2012
	14	0.0000	0.1980	0.6268	*0.8221*	0.5924	0.1923
	21	0.0311	0.2539	0.5440	*0.6437*	0.4593	0.1563
	28	0.0000	0.0913	0.3100	0.5371	*0.6400*	0.2947
	40	0.0000	0.0000	0.0902	0.4151	*0.8151*	0.5244

IVCO 4	0	0.0000	0.2071	0.5883	*0.9461*	0.8608	0.3136
	7	0.0000	0.2058	0.6469	*0.8294*	0.5850	0.1864
	14	0.0000	0.1842	0.5972	*0.8131*	0.6043	0.2012
	21	0.0000	0.4080	*1.0790*	0.9622	0.3328	0.0000
	28	0.0000	0.4521	*1.1041*	0.9539	0.3231	0.0000
	40	0.0000	0.4220	*1.0759*	0.8993	0.3733	0.0380

IVCO 5	0	0.0000	0.2245	0.6912	*0.8484*	0.5686	0.1711
	7	0.0000	0.1942	0.6215	*0.8222*	0.5961	0.1945
	14	0.0000	0.1847	0.6003	*0.8153*	0.6044	0.2012
	21	0.0000	0.4178	*1.0951*	0.9580	0.3270	0.0000
	28	0.0000	0.4295	*1.1114*	0.9516	0.3206	0.0000
	40	0.0000	0.4270	*1.0846*	0.8983	0.3693	0.0373

### Validation of new developed equation against our previous finding by e-nose technology

The new developed equation for deriving defuzzified scores under condition (a + c) > 100, unambiguously concluded that VCO sample irradiated at 4.2 kGy on day 28 of storage was the best quality sample, in complete agreement with the findings of sensory evaluation and e-nose technology [3].

From the above discussion, it can be concluded that the results of similarity value and the e-nose analyses were in agreement with that obtained by the new developed equation of fuzzy logic analysis. As per the new equation, the defuzzified scores were evaluated and it’s corresponding descriptions implied that IVCO 3 was regarded as ‘the best’ with respect to deodorization (quality attribute: aroma) which was effective from day 28 of storage Similarity value analysis also designated IVCO 3 as ‘very good’ in terms of deodorization in accordance with six point standard fuzzy scale. This conclusion was further proved by the e-nose signal responses (ΔR/R) of the irradiated coconut oil samples. This new developed equation can be widely adopted for ranking food samples unambiguously, rapidly and reliably, without any conflict with similarity value and e-nose approaches. It evades the complexity in evaluating defuzzified scores of samples, compared to use of similarity values.
